# Effective anti-doping testing program during major sport events: a comparative analysis of two multi-sport tournaments held in Poland in 2017 and 2023

**DOI:** 10.3389/fspor.2025.1628088

**Published:** 2025-08-11

**Authors:** Elżbieta Lipska, Katarzyna Szamotulska, Dorota Kwiatkowska, Michał Rynkowski

**Affiliations:** ^1^ Endocrinology Outpatient Clinic, Institute of Mother and Child, Warsaw, Poland; ^2^ Department of Epidemiology and Biostatistics, Institute of Mother and Child, Warsaw, Poland; ^3^ Polish Anti-Doping Laboratory, Warsaw, Poland (PLAD); ^4^ Biological and Chemical Research Centre, University of Warsaw, Warsaw, Poland; ^5^ Polish Anti-Doping Agency, Warsaw, Poland (POLADA)

**Keywords:** anti-doping program, multi-sports tournament, doping testing, major sporting event, mass gathering medicine

## Abstract

**Introduction:**

Major Sporting Events (MSEs), particularly Multi-Sport Tournaments (MSTs), present significant logistical and operational challenges in the implementation of effective Anti-Doping Programs (ADPs). This study presents a comparative analysis of ADP implementation during two MSTs hosted in Poland: The World Games 2017 (TWG 2017) and the European Games 2023 (EG 2023).

**Methods:**

The analysis encompasses organizational logistics, sample collection processes, laboratory testing, and the broader implications for national anti-doping activities conducted by the Polish Anti-Doping Agency (POLADA) and the Polish Anti-Doping Laboratory (PLAD), which in 2017 operated under the name Department of Anti-Doping Research at the Institute of Sport.

**Results:**

TWG 2017 involved 3,292 athletes, whereas EG 2023 hosted 6,380 participants. Although both events utilized the same number of sport venues, the wider geographical distribution of EG 2023 introduced greater logistical complexity. The number of Sample Collection Personnel (SCP) increased in absolute terms for EG 2023, comprising 90 Doping Control Officers (DCO) and 120 chaperones, in contrast to 37 DCOs and 35 chaperones during TWG 2017. However, when adjusted to the number of athletes, the increase was not statistically significant. This rise in SCP corresponded to a significant increase in testing volume: 1,210 samples were collected at EG 2023 compared to 401 at TWG 2017, with a notably higher proportion of out-of-competition tests. Both MSTs placed considerable demands on the routine operations of POLADA and PLAD.

**Discussion:**

Despite structural similarities and the involvement of shared institutional stakeholders, EG 2023 represented a significant scale-up in ADP due to the increased number of athletes, greater geographical dispersion, and enhanced testing complexity. These findings underscore the necessity for scalable, context-specific and coordinated anti-doping strategies. These need to be tailored to the unique operational demands of MSTs, distinguishing them from single-sport events.

## Introduction

Major Sporting Events (MSEs) involve a complex and extensive organizational framework, engaging numerous stakeholders across various sectors. This organizational challenge becomes more intricate in the case of major Multi-Sport Tournaments (MSTs), which encompass multiple disciplines, diverse venues—spread across cities and regions—and the involvement of numerous institutions.

One of the primary organizational areas relevant to MSTs is the healthcare system, which encompasses the Anti-Doping Program (ADP)—a critical component for ensuring athlete safety and preserving the integrity of sport (1–4). As doping represents a significant threat to both fair play in sport and public health, the implementation of an effective ADP is essential. The ADP is embedded in the World Anti-Doping Code (1), and is further developed by the World Anti-Doping Agency (WADA) 2023 Guidelines for Major Events (2) and the WADA 2021 Guidelines for Implementing an Effective Testing Program (5). In this context, Testing Distribution Plans (TDPs) are developed by the Main Event Organization—the ruling body for the MSE—based on the annually updated WADA Technical Document for Sport-Specific Analysis (6, 7).

A significant challenge for ADPs in MSTs—compared to single-sport MSEs—is the time sensitivity involved. Processing of samples at a WADA-accredited laboratory is essential to ensure results availability before subsequent competition stages (8, 9). The complexity of ADPs increases when events are hosted simultaneously at geographically dispersed venues (8).

Sample Collection Personnel (SCP) involved in the ADP include highly trained professionals, such as medical staff serving as Doping Control Officers (DCO) and Blood Control Officers (BCO) (2). They are recruited from National Anti-Doping Organizations (NADOs), third-party medical institutions, and internationally to ensure sufficient staffing levels and effective communication with athletes during the doping control process. Additionally, volunteers are recruited as chaperones, supporting the SCP. All SCPs must be legally authorized to conduct medical procedures and must also receive event-specific training tailored to the unique demands of the MSE and ADP (2).

SCPs perform their responsibilities during assigned testing missions. Two types of missions exist: (1) DCO missions, focused on urine samples collection; and (2) joined DCO and BCO missions, aimed at collecting urine and blood samples (2). Each mission is supported by chaperones, who assist with the implementation of testing protocols at the venue.

Since co-hosting the UEFA EURO 2012 Football Championship with Ukraine—the first MSE of such scale held in Poland—the country has served as host to numerous other international competitions, including two MSTs: The World Games 2017 in Wrocław (TWG 2017) and The European Games 2023 in Kraków and the Małopolska Region (EG 2023). The primary anti-doping authorities responsible for both MSTs were the Polish National Anti-Doping Organization (POLADA) and the Polish Anti-Doping Laboratory (PLAD), which, in 2017, operated under the name Department of Anti-Doping Research at the Institute of Sport.

This study undertakes a comparative analysis of the organization of ADPs implemented during the two aforementioned MSTs, with particular emphasis on the structure of the testing programs, the volume of samples collected and tests conducted, as well as the operational workload borne by anti-doping personnel. In light of the extensive medical planning required for MSTs and the constraints posed by limited resources, the deployment of accurate and efficient staffing strategies is essential. Additionally, this analysis encompasses a comparative assessment of the scale of ADPs executed during these MSTs relative to the routine activities carried out by the POLADA and the PLAD in 2017 (10, 11) and 2023 (12), respectively.

## Materials and methods

A comparative analysis was conducted on two multi-sport tournaments held in Poland: The World Games 2017 and The European Games 2023. TWG 2017 was a global tournament featuring non-Olympic sports, with participation from 103 national teams and 3,292 athletes competing in 31 disciplines across 115 sporting events, held at 26 venues over 11 tournament days. EG 2023, a regional tournament for European nations, included several Olympic qualification events and featured 49 national teams and 6,380 athletes competing in 29 disciplines across 134 sporting events, also held at 26 sport venues over 13 tournament days. General and sport-specific data are presented in Table 1.

**Table 1:** Comparison of general and sport-specific data: The World Games 2017 & European Games 2023.

Category	The World Games 2017	European Games 2023
General information		
Main Event Organizer	International World Games Association (IWGA)	European Olympic Committees (EOC)
Duration of Tournament	11 days	13 days
Host Cities	1 (Wrocław, Poland)	14 (Kraków and Małopolska Region, Poland)
Number of Venues	26	26
Venue Capacities	Ranging from 150 to 42,000 spectators	Ranging from 500 to 14,999 spectators
Distance Between Venues	42 kilometers	495 kilometers
Total Spectator Attendance	Approximately 240,000	Approximately 172,000
Sport-specific data		
Number of Participating National Teams	103	49
Number of Athletes	3,292	6,380
Female Athletes	1,324 (40.2%)	3,095 (48.5%)
Male Athletes	1,968 (59.8%)	3,285 (51.5%)
Number of Disciplines	32	29
Number of Sport Events	115	135

TWG 2017 was hosted in the city of Wrocław, with venues extending up to 42km from the city center. In contrast, EG 2023 was held in Kraków and 13 additional locations across the Małopolska Region and other cities such as Rzeszów and Wrocław, with the distance between venues reaching 495km. The ADPs at both events were conducted by the POLADA, supervised respectively by International World Games Association, International Testing Agency and third-party local medical organizations. No structured pre-tournament testing programs were implemented at either event.

During the tournament phase, TWG 2017 involved 72 SCPs across 69 testing missions. The SCP included 10 international DCOs from Barbados, Belarus, Czechia, South Africa, Slovakia and the United Kingdom. In comparison, EG 2023 involved 210 SCPs, including 43 international DCOs from Armenia, Austria, Estonia, France, Georgia, Greece, Israel, Italy, Kazakhstan, the Netherlands, Norway, Romania, Slovenia, Sweden, Switzerland, Ukraine, the United Kingdom. A total of 236 testing missions were conducted.

Across both MSTs, a total of 1,611 samples were collected during in-competition (IC) and out-of-competition (OOC) periods. All samples were analyzed at the PLAD in Warsaw, resulting in 2,316 tests performed. Additionally, 75 dried blood spot (DBS) samples collected during EG 2023 were analyzed at the Anti-Doping Laboratory in Cologne. The reporting time was standard during TWG 2017, whereas expedited reporting was required during EG 2023.

Adverse analytical findings (AAFs) were identified in four athletes during TWG 2017 (13); no AAFs were reported during EG 2023 (14). The data for this study was obtained from TDPs and ADPs (internal documents), official final tournament reports (15), and observers documentation (8, 16, 17).

Statistical analysis: A retrospective comparative analysis was conducted using descriptive statistical methods. In addition, the chi-square test and the chi-square test for proportions were applied. A *p*-value of less than 0.05 was considered statistically significant.

## Results

The results encompass the sporting profile, organization and geographical characteristics of the MSTs, deployment of SCP, number and distribution of collected samples according to TDPs, gender distribution of samples, laboratory testing activity, and a comparative assessment of ADPs and laboratory workload relative to the annual operations of POLADA and PLAD in 2017 and 2023, respectively.

### Sporting profile

Both TWG 2017 and the EG 2023 were major multi-sport tournaments, differing notably in scale and gender distribution among participants. TWG 2017 featured a total of 3,292 athletes, comprising 1,968 men (59.8%) and 1,324 women (40.2%). In contrast, EG 2023 nearly doubled the number of participants, hosting 6,380 athletes with a more balanced gender distribution: 3,285 men (51.5%) and 3,095 women (48.5%).

The observed male-to-female imbalance during TWG 2017 was primarily due to the inclusion of four male-only disciplines—American football, fistball, floorball, and speedway—which collectively featured 345 athletes, compared to only one female-only discipline, lacrosse, with 90 athletes. Additionally, several disciplines exhibited a male-to-female athlete ratio exceeding 70:30, including air sports, billiards, muaythai, and roller sports. Fourteen disciplines had a nearly equal distribution of male and female athletes, while in remaining five, a moderate male predominance was observed.

In contrast, during EG 2023, 15 disciplines were gender-balanced, 13 showed male predominance, and one (artistic swimming) demonstrated a strong female predominance (94.7%). Notably, no single-gender disciplines were present.

Despite the higher number of athletes at EG 2023, TWG 2017 featured a greater number of national teams—103 compared to 49 at EG 2023. The number of sporting disciplines was comparable, with 31 at TWG 2017 and 29 at EG 2023. Similarly, the total number of sport events was comparable: 115 and 134, respectively.

### Organization and geographical distribution

While both tournaments utilized 26 sports venues, their geographical layouts differed markedly. TWG 2017 was concentrated within a single city and its suburbs—Wrocław—while EG 2023 was distributed across 14 different locations within the Małopolska Region and in cities Wrocław and Rzeszów. This broader geographical dispersion in EG 2023 posed increased logistical challenges for the implementation of the ADP, particularly in terms of the number of testing missions and personnel required.

### Sample collection personnel

The composition and scale of the SCP teams varied between the two events. At TWG 2017, the SCP team consisted of 37 professional control officers, including 27 DCOs, 10 BCOs, supported by 35 chaperones. This staffing level resulted in an average of one control officer per 89 athletes, one DCO per 122 athletes, one BCO per 329 athletes, and one chaperone per 94 athletes.

In contrast, EG 2023 deployed a larger SCP team comprising of 90 professional control officers, including 60 DCOs, 30 BCOs, alongside 120 chaperones. This resulted in an average of one control officer per 71 athletes, one DCO per 106 athletes, one BCO per 213 athletes, and one chaperone per 53 athletes.

A comparison of the SCP-to-athlete ratios—that is, the number of SCP personnel per athlete—between TWG 2017 and EG 2023 revealed no statistically significant differences, except when chaperones were included. In that case, the differences became statistically significant, whether chaperones were considered independently or in combination with COs. Detailed data are presented in Table 2.

**Table 2:** Summary of statistical comparisons between TWG 2017 and EG 2023.

Category	*χ*² (df)*		*p*-value
Sample collection personnel to athletes (TWG 2017 vs. EG 2023)			
Control Officers	1.13 (1)		.287
Doping Control Officers	0.22 (1)		.635
Blood Control Officers	1.07 (1)		.299
Chaperones	8.43 (1)		**.** **003**
Control Officers and Chaperones	8.48 (1)		**.** **003**
Samples (TWG 2017 vs. EG 2023)			
In-competition to Out-of-competition			
Total	155.59 (1)		**<** **.** **001**
Urine	101.22 (1)		**<** **.** **001**
Blood	82.42 (1)		**<** **.** **001**
Blood to Urine	1.31 (1)		.251
Sample to athletes			
Total	52.04 (1)		**<**.**001**
Urine	51.31 (1)		**<**.**001**
Blood	2.79 (1)		.094
Total Out-of-competition	129.20 (1)		**<**.**001**
Total In-competition	0.0002 (1)		.988
Control officers workload			
Total Samples	0.91 (1)		.340
Urine to Doping Control Officer	1.60 (1)		.204
Blood to Blood Control Officer	0.05 (1)		.814
Control officers and chaperones workload			
Total Samples	0.02 (1)		.877
Urine	0.11 (1)		.737
Blood	1.18 (1)		.275
Testing missions			
Total	2.15 (1)		0.142
Total (chaperones included)	0.52 (1)		0.466
Blood	0.09 (1)		0.760
Blood (chaperones included)	1.18 (1)		0.276
	group proportions	z**	*p*-value
Samples gender distribution			
Gender (TWG 2017)	F (*n*_1_ = 1,324, *p*_1_ = .123) M (*n*_2_ = 1,968, *p*_2_ = 0.121)	0.19	0.851
Gender (EG 2023)	F (*n*_1_ = 3,095, *p*_1_ = 0.184) M (*n*_2_ = 3,285, *p*_2_ = 0.195)	−1.15	.251
Female Athletes	TWG 2017 (*n*_1_ = 1,324, *p*_1_ = 0.123) EG 2023 (*n*_2_ = 3,095, *p*_2_ = 0.184)	−4.97	**<0**.**001**
Male Athletes	TWG 2017 (*n*_1_ = 1,968, *p*_1_ = 0.121) EG 2023 (*n*_2_ = 3,285, *p*_2_ = 0.195)	−6.97	**<0**.**001**
	χ² (df)*		*p*-value
Laboratory tests (TWG 2017 vs. EG 2023)			
In-competition to out-of-competition			
Urine	167.33 (1)		**<0**.**001**
Blood	91.47 (1)		**<0**.**001**
Total	293.58 (1)		**<0**.**001**
Number of tests per sample			
Urine	0.3 (1)		0.583
Blood	0.003 (1)		0.953
Total	0.16 (1)		0.683
Number of tests per athlete			
Urine	59.43 (1)		**<0** **.** **001**
Blood	6.01 (1)		**0** **.** **014**
Total	61.52 (1)		**<0**.**001**

Bold values indicate statistical significance.

* Yates’ correction applied.

** Two-proportion z-tests were used to compare testing rates. Significance threshold set at *p* < .05.

**Figure 1:**
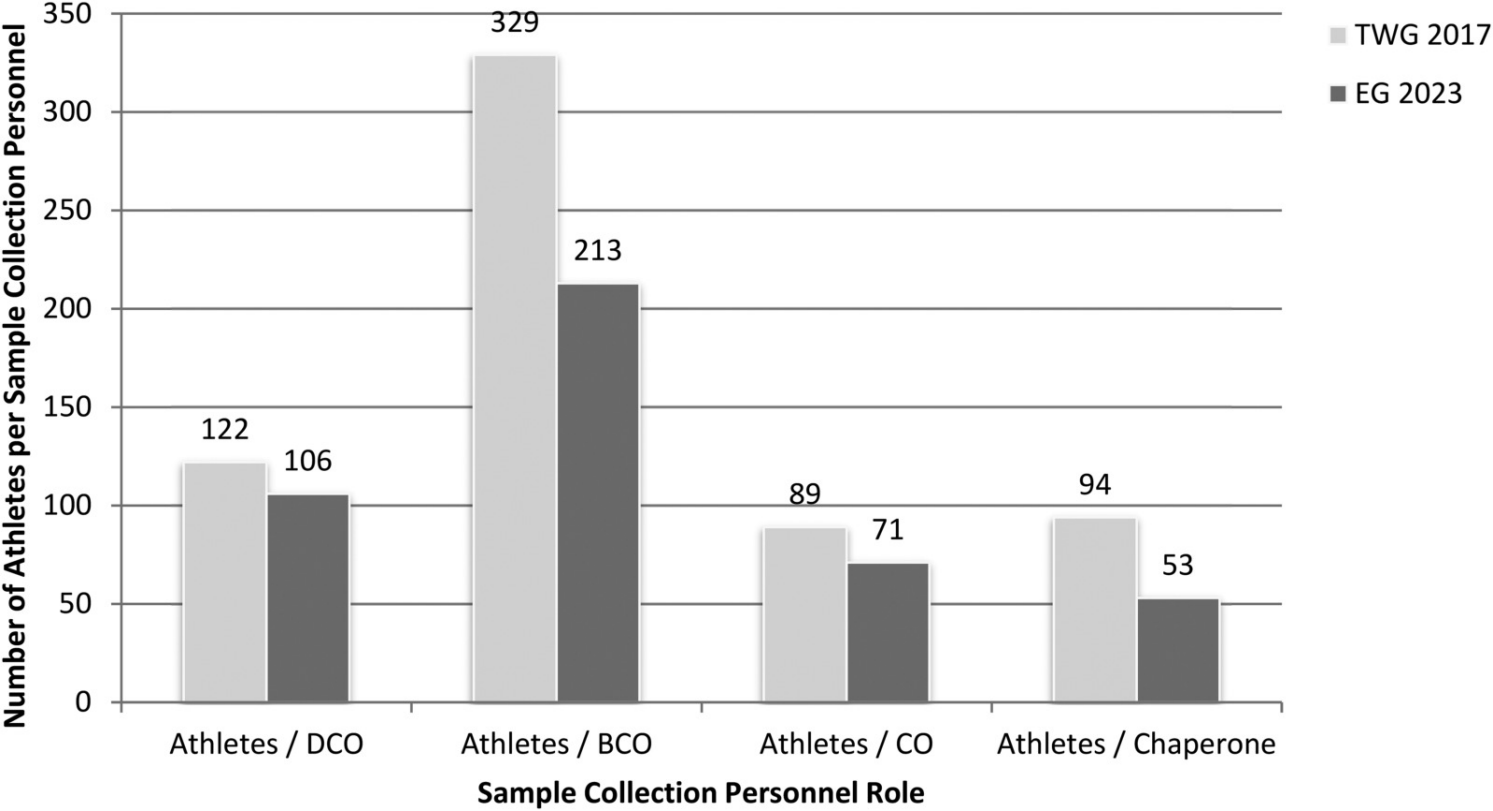
Athlete-to-sample collection personnel ratios at The World Games 2017 (TWG 2017) and European Games 2023 (EG 2023). DCO, doping control officer; BCO, blood control officer; CO, control officer.

Figure 1 illustrates the proportional relationship between the number of SCPs and the number of participating athletes in TWG 2017 and EG 2023.

### Samples

Samples were collected in both IC and out-of-competition OOC settings at both tournaments. At TWG 2017, a total of 401 samples were collected, averaging one sample per 8.2 athletes, corresponding to 12.2% participants. Of these, 24 (6.0%) were collected OOC and 377 (94.0%) were collected IC. In comparison, EG 2023 yielded a significantly higher total of 1,210 samples, averaging one sample per 5.3 athletes, or 18.9% participants. Of these, 477 (39.4%) were OOC samples and 733 (60.6%) were IC samples.

Urine samples constituted the majority of collected samples. At TWG 2017, 341 samples were obtained (one per 9.5 athletes), with 21 (6.2%) taken OOC and 320 (93.8%) collected IC. EG 2023 involved the collection of 1,058 urine samples (one sample per 6 athletes), with 363 (34.3%) taken OOC and 695 (65.7%) IC.

Blood samples represented a smaller, but essential part of the testing program. At TWG 2017, 60 blood samples were collected (one per 54.9 athletes), with 3 (5%) collected OOC and 57 (95%) collected IC. EG 2023 reported a higher volume, with 152 blood samples collected—114 (75%) OOC and 38 (25%) IC. Additionally, 75 DBS samples were collected during EG 2023.

A comparison of in-competition (IC) and out-of-competition (OOC) testing between TWG 2017 and EG 2023 revealed a statistically significant difference in the total number of tests, and separately for urine and blood tests. However, no significant difference was found in the distribution of blood and urine sample (Table 2).

A comparison of the total number of samples collected per athlete also showed a statistically significant difference, as did the comparison of urine tests and total OOC tests. In contrast, no statistically significant differences were observed for blood tests or total IC tests (Table 2).

Regarding control officers workload, at TWG 2017, the average number of total samples collected per control officer was 10.8, comprising 12.6 urine samples per DCO and 6 blood samples per BCO. In contrast, EG 2023 showed a higher control officers workload, with an average of 13.4 total samples per control officer, including 17.6 urine samples per DCO and 5.1 blood samples per BCO (excluding DBS samples). No statistically significant differences were observed in the workload of control officers and chaperones between the two tournaments, regardless of whether chaperones were included.

A total of 69 testing missions were conducted at TWG 2017, equating to an average of 1.86 missions per CO. Of these, 37 were blood-collection missions, resulting in an average of 3.7 missions per BCO. EG 2023 involved a substantially larger testing program, with 236 anti-doping missions in total—averaging 2.6 missions per CO. This included 90 blood-collection missions, with an average three missions per BCO. Also here, no statistically significant differences were observed in the workload of COs and chaperones between the two tournaments, regardless of whether chaperones were included (Table 2).

**Figure 2:**
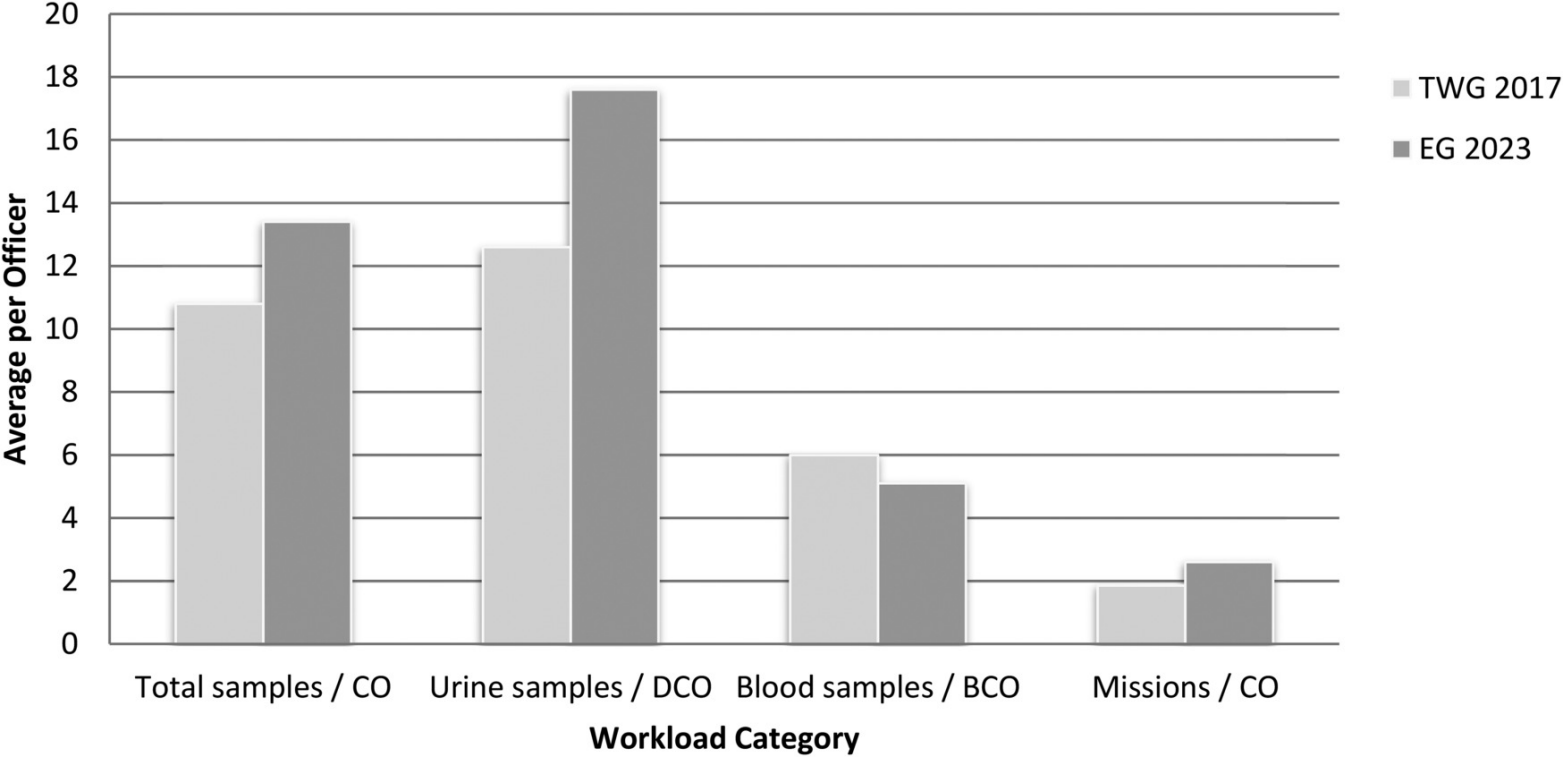
Comparative workload of control officers during TWG 2017 and EG 2023. CO, control officer; DCO, doping control officer; BCO, blood control officer.

Figure 2 presents a comparative overview of control officers workload during TWG 2017 and EG 2023.

### Samples gender distribution

In terms of gender distribution, TWG 2017 recorded the collection of 163 samples from female athletes (12.3%) and 238 from male athletes (12.1%). At EG 2023, the number of samples increased substantially, with 569 samples collected from female athletes (18.4%) and 641 from male athletes (19.5%).

Urine samples distribution at TWG 2017 included 144 samples (10.9%) from female athletes and 197 samples (10.0%) from male athletes. In comparison, EG 2023 recorded 511 urine samples (16.5%) from female athletes and 546 (16.6%) from male athletes. Blood samples at TWG 2017 were collected from 19 female athletes (1.4%) and 41 male athletes (2.0%). During EG 2023, 59 blood samples (1.9%) were collected from female athletes and 94 (2.8%) from male athletes.

There were no statistically significant differences in the gender distribution of samples within each tournament or between them. However, there was a statistically significant increase in the testing rate from TWG 2017 to EG 2023 for both male and female athletes (Table 2).

### Laboratory tests

All samples were analyzed at the PLAD. Both urine and blood tests included panels of multiple parameters. At TWG 2017, 30 tests were conducted on 21 out-of-competition urine samples, yielding an average of 1.42 tests per sample. In-competition urine samples accounted for 441 tests across 320 samples, with an average of 1.38 tests per sample. For blood testing six tests were conducted on three out-of-competition blood samples and 114 tests on 57 in-competition blood samples, with both groups averaging 2.0 tests per sample.

At EG 2023, 550 tests were performed on 363 out-of-competition urine samples, with an average of 1.51 tests per sample, and 869 tests on 695 in-competition urine samples, averaging 1.25 tests per sample. In terms of blood analysis, 230 tests, including 45 related to the Athlete Biological Passport (ABP), were conducted on 114 out-of-competition blood samples, with an average of 2.0 tests per sample. A further 76 tests, including one ABP test, were conducted on 38 in-competition blood samples, averaging 2.0 tests per sample.

In the comparison between both tournaments, there is a statistically significant difference in IC and OOC testing for urine, blood and total tests, but not in the number of tests per sample (Table 2).

Overall, during TWG 2017, 471 urine tests were conducted across 3,292 athletes, representing 14.3% of participants, while 120 blood tests were performed, covering 3.6% of athletes. In contrast, EG 2023 saw a total of 1,419 urine tests across 6,380 athletes (22.2%) and 306 blood tests (4.8%). There is a statistically significant difference in the number of tests per athlete in urine, blood and the total number of tests between TWG 2017 and EG 2023 (Table 2).

**Figure 3:**
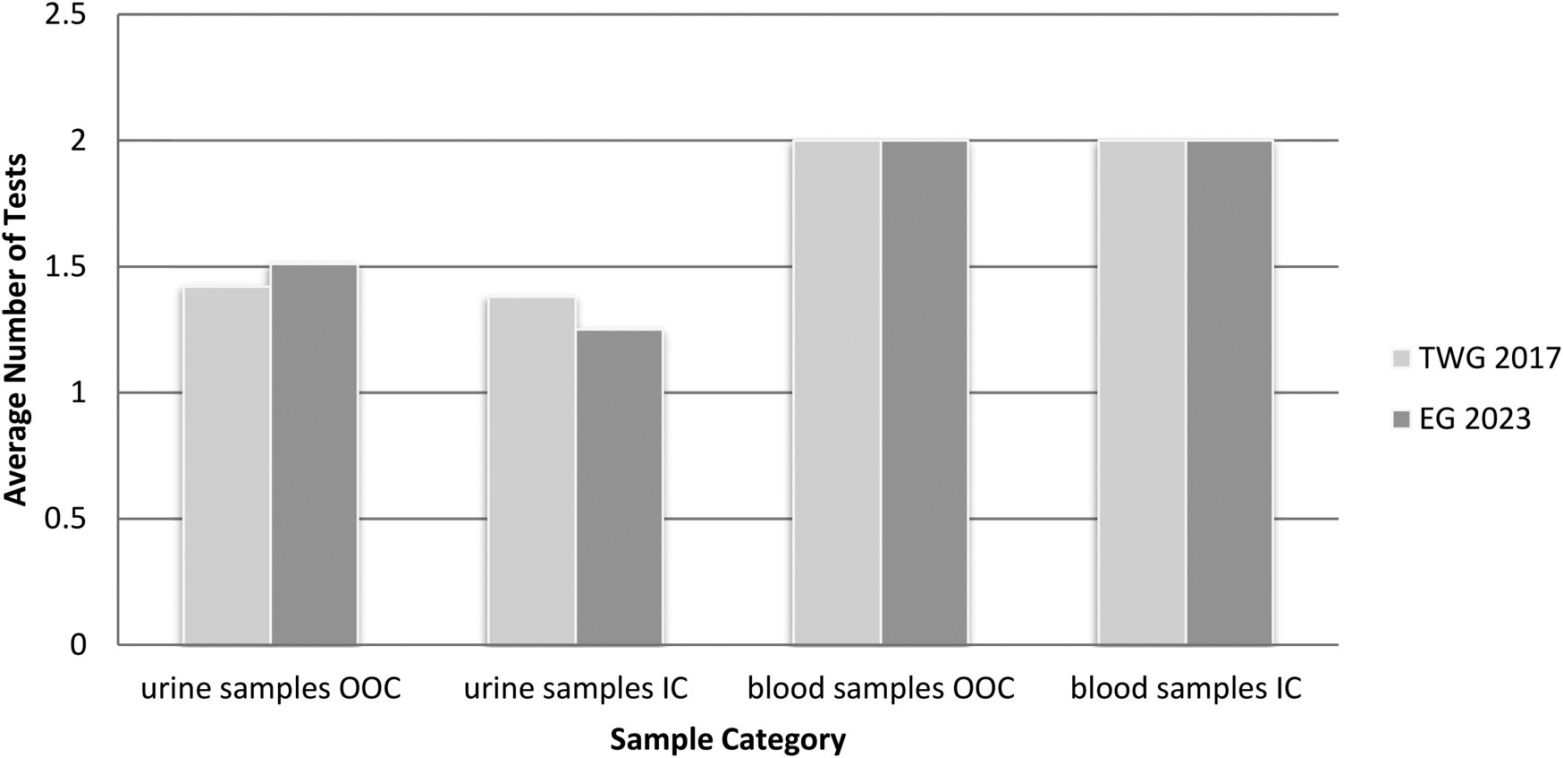
Comparison of the average number of PLAD laboratory tests per sample conducted during TWG 2017 and EG 2023. OOC, out-of-competition; IC, in-competition.

Figure 3 presents PLAD laboratory tests per sample during TWG 2017 and EG 2023.

### MSTs in comparison to the annual activities of POLADA and PLAD

**Figure 4:**
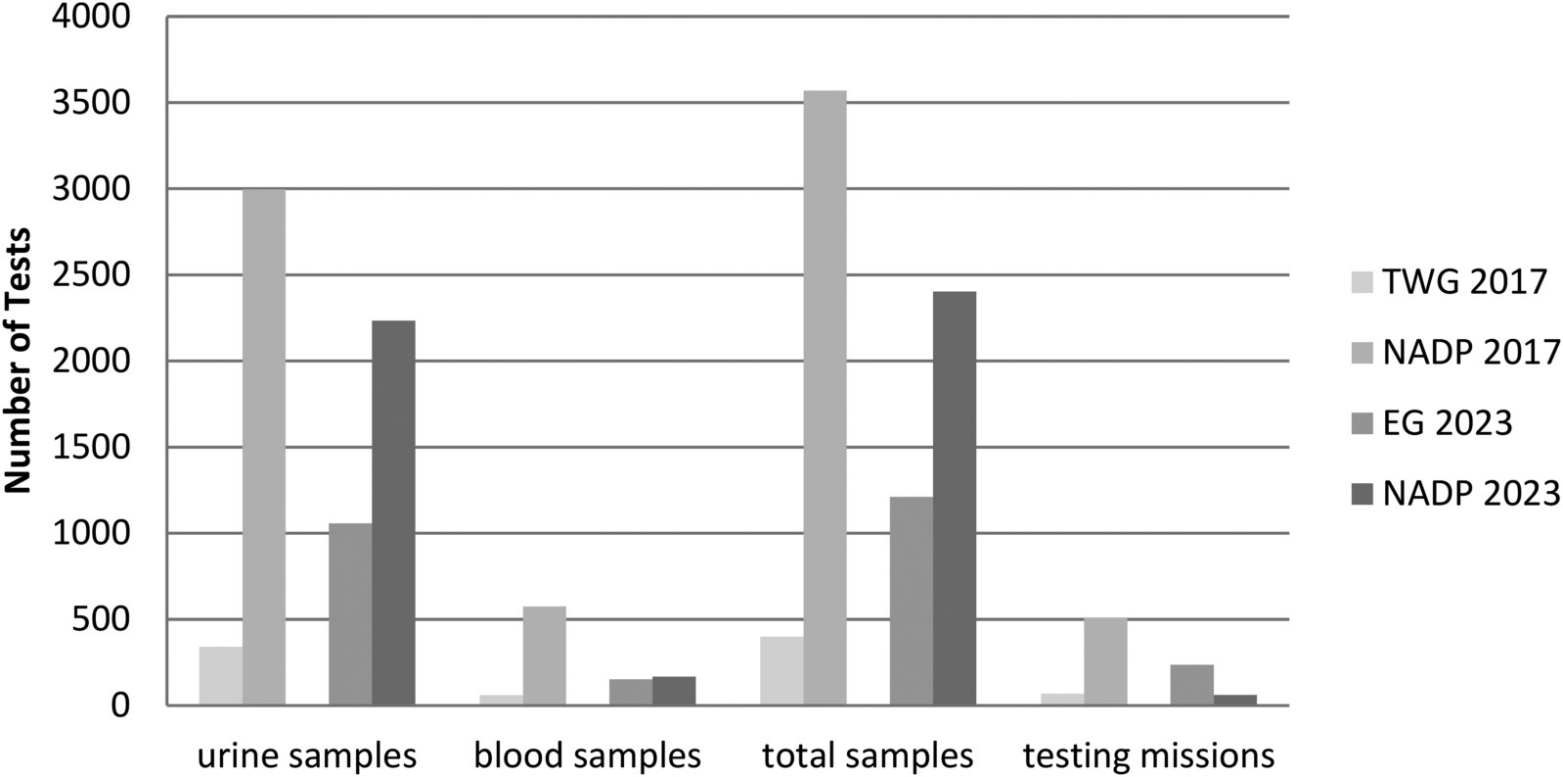
Comparative analysis of TWG 2017 and EG 2023 testing activities relative to the national anti-doping plans (NADP) implemented by POLADA in 2017 and 2023. NADP, national anti-doping plan; POLADA, Polish anti-doping agency.

The impact and scale of TWG 2017 and EG 2023 were further evaluated in relation to the broader anti-doping activities undertaken by POLADA and PLAD within their respective years. As presented in Figure 4, TWG 2017 and EG 2023 were significant events compared to the annual testing plans in Poland. Despite their relatively short durations, both tournaments accounted for substantial portions of the total anti-doping activities conducted by POLADA and PLAD, highlighting the organizational scale and operational intensity required to support anti-doping programs during major multi-sport tournaments.

During TWG 2017, the ADP conducted over the 11-day event accounted for 11.4% of all urine samples collected in 2017, 10.4% of blood samples, and 11.2% of total samples, while also representing 13.5% of the testing missions carried out by POLADA that year. This figures highlight that, despite the relatively short duration, TWG 2017 resembled a notable portion in comparison with national testing program.

In contrast, EG 2023 had a far greater impact on the national anti-doping landscape. Over its 13-day span, 47.4% of urine samples, 91.1% of blood samples, and 50.4% of total samples were collected, compared to the total samples taken across the entire year. Perhaps more significantly, the number of testing missions was 3.8-fold higher compared to the annual average, underscoring the exceptional intensity and resource demands of the testing program implemented during EG 2023.

Figure 4 illustrates the proportion of TWG 2017 and EG 2023 testing activities in comparison to the overall National Anti-Doping Plans (NADPs) executed by POLADA in 2017 and 2023, respectively.

**Figure 5:**
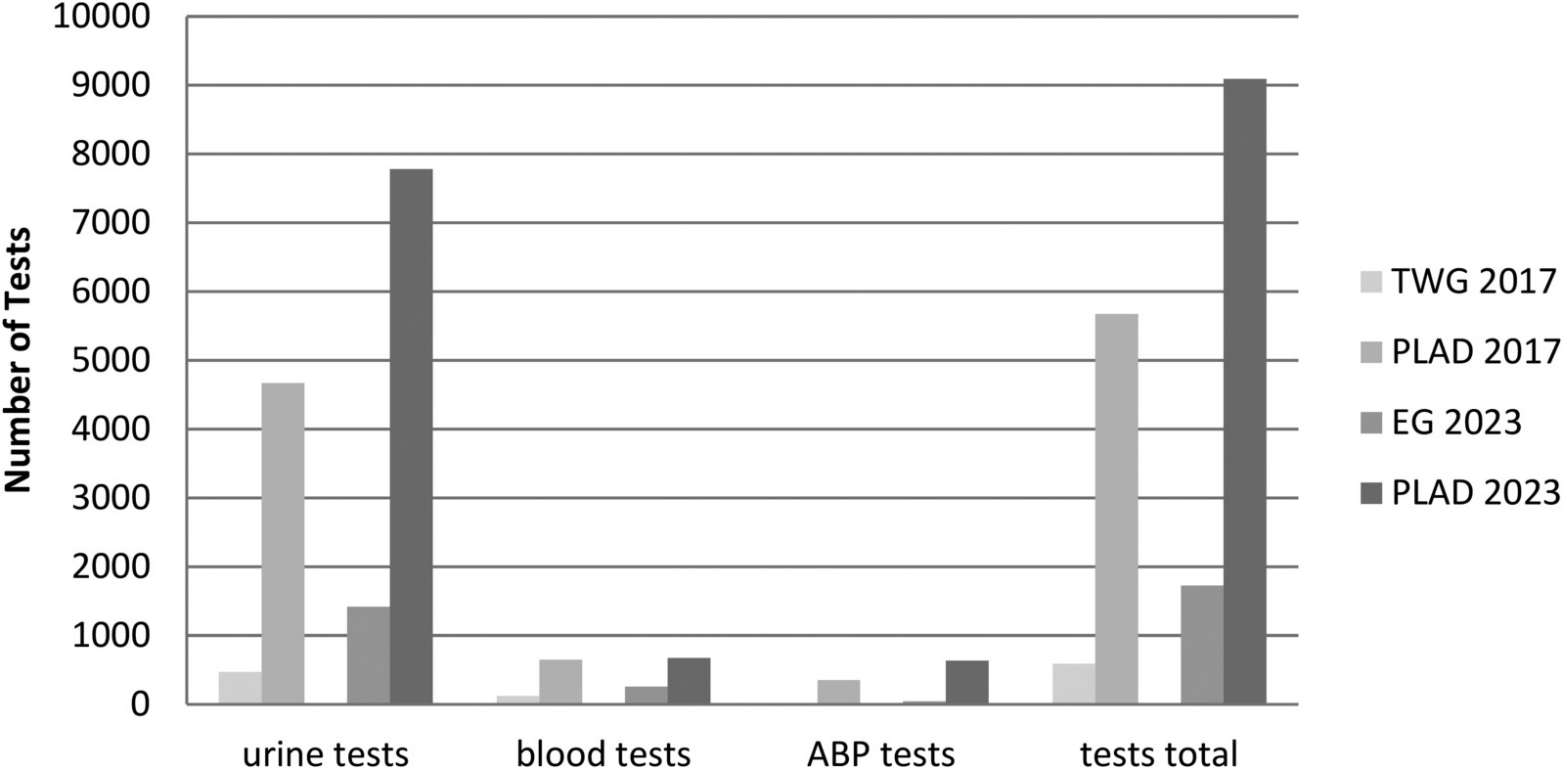
Analysis of TWG 2017 and EG 2023 laboratory testing contribution relative to Polish anti-doping laboratory (PLAD) annual workload in 2017 and 2023. ABP, athlete blood passport; PLAD, Polish anti-doping laboratory.

Analysing PLAD's annual samples analyses, TWG 2017 represented 10.0% of all urine tests, 18.4% of blood tests, no ABP tests, and 10.4% of the total tests conducted by PLAD in 2017. In comparison, EG 2023 accounted for 18.2% of urine tests, 31.9% of blood tests, 7.1% of ABP tests, and 18.9% of all tests carried out by PLAD in 2023. Figure 5 illustrates the proportion of TWG 2017 and EG 2023 laboratory testing activities in relation to PLAD's yearly workload for the years 2017 and 2023, respectively.

## Discussion

Organizing a major multi-sport tournament presents inherent complexity and operational challenges (18). TWG 2017 and EG 2023 shared numerous structural similarities, including the number of venues, event duration, number of disciplines, and total number of sporting events. However, key differences emerged, particularly regarding the number and composition of participating national teams. While TWG 2017 hosted twice as many national delegations, EG 2023 accommodated twice the number of athletes. This suggests that TWG 2017 attracted a larger number of smaller national teams, whereas EG 2023 involved fewer but more populous delegations. Another significant distinction was the geographic dispersion of EG 2023 venues, which posed considerable logistical challenges in contrast to TWG 2017, localized within a single city.

### Staffing models

The scale and complexity of the ADPs at both events required a high level of institutional preparedness and effective cross-agency coordination. A consistent strength of both ADPs was the involvement of the same core institutions: the Polish Anti-Doping Agency (POLADA) and the Polish Anti-Doping Laboratory (PLAD). The participation of a professional, experienced, and cohesive team contributed substantially to the effective implementation of both ADPs. A notable feature was the extensive involvement of medical personnel, particularly in the role of blood control officers (BCOs), which consequently reduced their availability for other medical duties during the tournaments and for regular healthcare services (19). This constraint should be addressed in broader medical planning for future events.

Both events demonstrated significant international cooperation, with doping control officers (DCOs) from other national anti-doping organizations (NADOs) providing essential operational support. At TWG 2017, 10 DCOs from 6 NADOs were engaged, whereas EG 2023 involved 43 DCOs from 17 NADOs. This collaboration not only strengthened implementation capacity but also served as a valuable platform for knowledge exchange and capacity-building in preparation for future MSTs. Another noteworthy example of international involvement was the DBS analysis conducted at the Anti-Doping Laboratory in Cologne.

The scale of sample collection personnel (SCP) at EG 2023 was approximately 2.4 times greater than at TWG 2017, with DCOs increasing by 2.2 times, BCOs by 3.0 times, and chaperones by 3.4 times. While the SCP-to-athlete ratios were higher across all categories at EG 2023, these differences were statistically significant only for chaperones. Moreover, the relative increases in SCP-to-athlete ratios were less pronounced than the overall growth in absolute staff numbers, ranging from 1.12 to 1.56 for control officer groups, and 1.9 for chaperones. These discrepancies likely reflect the increased complexity of the testing strategy, the logistical demands of geographically dispersed venues, and scheduling overlaps. The contribution of trained chaperones should be emphasized, as their support enhanced the operational efficiency of the ADP and helped alleviate the burden on core anti-doping personnel.

In contrast, single-sport major sporting events (MSEs) typically require fewer SCP. For example, during the in-competition phase of UEFA EURO 2012, the anti-doping team comprised only 6 DCOs, 6 BCOs, and 32 chaperones—corresponding to a ratio of one control officer per 31 players, and one chaperone per 11 players (20). Despite the high profile of the tournament (21), both the numbers of players and SCP were markedly lower than those required for MSTs. Similar trends were observed in other events, such as the EHF EURO 2016 and the 2014 FIFA World Cup (9, 22).

### Venue logistics

Sample collection at EG 2023 was not only more complex than at TWG 2017 but also demonstrably more effective. Athletes were tested 1.5 times more frequently at EG 2023 (18.9% vs. 12.2%). The Test Distribution Plan (TDP) was more balanced and aligned with international standards, comprising 60.6% in-competition (IC) and 39.4% out-of-competition (OOC) samples. At TWG 2017, 94.1% of samples were collected during IC testing, indicating a less comprehensive approach.

Despite the gender imbalance among athletes at TWG 2017—primarily due to single-gender disciplines—the TDP remained equitably structured in line with WADA requirements. At EG 2023, slightly more samples (1.1%) were collected from male athletes; however, this difference was not statistically significant and was observed only in blood samples.

The differences observed in the ADPs reflected the structural distinctions of the respective MSTs. TWG 2017, featuring exclusively non-Olympic disciplines, focused more on education and outreach activities. Conversely, EG 2023 included Olympic disciplines and Olympic qualification events, thereby requiring more rigorous and extensive testing program.

The testing strategy at EG 2023 aligned with models implemented during the Rio 2016 and Paris 2024 Olympic Games, which featured IC to OOC ratios of 59:41 and 66:34, respectively (23–25). Nonetheless, overall testing levels at EG 2023 were understandably lower than those implemented at the Olympic Games. For instance, during Paris 2024, 6,130 samples were collected from 10,714 athletes, with a testing coverage of 38.75% (52% male, 48% female), and testing ratios of one sample per 1.2 athletes, one urine sample per 1.37 athletes, and one blood sample per 9.4 athletes (24–26). Similar metrics were reported at the Tokyo 2020 Olympic Games (27).

Historical data demonstrate even greater gender disparities. For example, at the 2011 World Athletics Championships in Daegu, the male-to-female testing ratio was 53% to 47% (28). In single-sport events such as UEFA EURO 2012, testing intensity was higher, with 320 OOC and 248 IC samples collected from 367 players—equating to over 1.5 samples per player (20).

### Laboratory workload

Analytical workload at WADA-accredited PLAD laboratory was comparable across both MSTs. Urine samples underwent between 1.24 and 1.51 tests per sample, with higher analytical frequencies for OOC samples (1.41–1.51) compared to IC samples (1.24–1.38). Blood samples were subjected to more extensive testing, averaging between 1.62 and 2.00 tests per sample. These differences were not statistically significant.

However, the number of tests per athlete was significantly higher at EG 2023 compared to TWG 2017. Urine testing increased by 22.1%, compared to 14.3% at TWG 2017, and blood testing rose by 4.1%, compared to 3.6%. A distinctive challenge at EG 2023 was the requirement for expedited reporting, which was not a factor in TWG 2017. These findings confirm the more complex and extensive nature of the testing program implemented at EG 2023.

For reference, during UEFA EURO 2012 in the same laboratory, 432 tests were conducted on 160 OOC urine samples (2.7 tests per sample) and 672 tests on 160 blood samples (5.4 tests per sample). During the IC phase, 468 tests were conducted on 124 urine samples (3.8 tests per sample) and 344 tests on 124 blood samples (2.8 tests per sample) (20).

### Adverse analytical findings and institutional impact

The absence of Adverse Analytical Findings (AAFs) during EG 2023, despite a sophisticated and wide-reaching ADP, is notable and may suggest the potential efficacy of clean sport strategies within a highly competitive sport environment. Nonetheless, these findings should be interpreted with caution. Limitations associated with intelligence-based ADPs and the volume of testing must be critically assessed. The current analysis provides additional insights that appear consistent with previous research indicating that higher testing volumes do not necessarily correlate with increased AAF detection (29–31).

In assessing the operational scale of ADPs at TWG 2017 and EG 2023, comparisons with the annual activities of POLADA and PLAD provide valuable insight. TWG 2017 accounted for approximately 10% of their annual workload, excluding blood testing, which represented 18.4% of PLAD total 2017 activity. In contrast, EG 2023 had a considerably greater impact, particularly in terms of sample collection. It accounted for nearly 50% of all urine samples and 91% of all blood samples collected under POLADA 2023 annual plan. Most notably, EG 2023 generated 4.2 times more testing missions than provided through the entire year. The testing workload for PLAD during EG 2023 was between 1.8 and 3.8 times higher than its annual activity, representing a significant increase compared to TWG 2017, though less pronounced than the increase observed for POLADA. Given that POLADA annual testing plan was comparable to those of other NADOs (29), these figures underscore the substantial organizational burden that MSTs place on national anti-doping agencies (30).

## Limitations

This study has several limitations that should be acknowledged. First, the analysis is primarily quantitative and operational in nature. While it provides a detailed account of testing volumes and logistical frameworks, it does not include formal qualitative data from key stakeholders involved in doping control programs during both MSTs. Second, while the findings are presented objectively, the authors' institutional affiliations with the anti-doping organizations involved in the events may introduce an element of subjective bias. Although no commercial or financial conflicts of interest exist, this potential bias has been noted to enhance the transparency of the study.

## Conclusions

This comparative analysis of anti-doping operations during The World Games 2017 and the European Games 2023 highlights the increasing complexity and scale of organizing anti-doping programs (ADPs) at multi-sport tournaments. Despite shared features such as event duration, structure, and the involvement of the same anti-doping organizations, the two events differed significantly in operational demands, particularly in terms of testing strategies.

The data confirm that while MSTs may feature lower sample-per-athlete ratios compared to single-sport events, their multidimensional nature demands broader operational planning, greater staffing, and extensive cross-agency collaboration. The sample-per-athlete ratio refers to the number of doping control samples collected divided by the number of athletes participating in the event. Although this ratio tends to be lower in MSTs due to the large and diverse athlete pool, the substantial burden imposed on national anti-doping agencies emphasizes the need for long-term institutional preparedness and scalable testing frameworks.

In light of the findings from this comparative analysis, several key recommendations can be proposed to support the effective implementation of anti-doping programs during future multi-sport events. Anti-doping programs should be planned well in advance and fully integrated into the overall organizational and medical planning of MSTs. Organizers should ensure that staffing models are systematically aligned with the specific demands of the testing strategy and are capable of adapting to the scale and complexity of the event. The implementation of structured and comprehensive training programs for all anti-doping personnel is essential to ensure a high standard of operational performance.

International collaboration should also be prioritized, including the involvement of personnel from other national anti-doping organizations. Such collaboration enhances implementation capacity, facilitates knowledge exchange, and supports the development of expertise necessary for the delivery of large-scale ADPs.

Testing strategies should be intelligence-led and based on a thorough risk assessment. A balanced approach between in-competition and out-of-competition testing is essential to meet international standards and ensure program credibility.

Equity in testing must be upheld across all athlete groups. Gender disparities in athlete representation should not result in inequitable testing practices.

Data generated during ADP implementation should be systematically collected and analyzed. Post-event evaluations should be conducted to assess program effectiveness, identify operational challenges, and inform future planning.

Finally, rigorous adherence to the World Anti-Doping Code must be upheld to ensure regulatory compliance and to support the ongoing evaluation and refinement of anti-doping strategies.

In conclusion, the insights derived from this analysis should inform future MST planning and policy development. A proactive, well-resourced, and collaborative approach to anti-doping will be critical to maintaining the integrity of sport at the multi-sport event level.

## Data Availability

The raw data supporting the conclusions of this article will be made available by the authors, without undue reservation.
